# Hurdle factors minimizing growth of *Listeria monocytogenes* while counteracting *in situ* antilisterial effects of a novel nisin A-producing *Lactococcus lactis* subsp. *cremoris* costarter in thermized cheese milks

**DOI:** 10.3934/microbiol.2018.1.19

**Published:** 2018-01-12

**Authors:** John Samelis, Athanasia Kakouri

**Affiliations:** Dairy Research Institute, General Directorate of Agricultural Research, Hellenic Agricultural Organization DEMETER, Katsikas, 45221 Ioannina, Greece

**Keywords:** *Listeria monocytogenes*, *Lactococcus lactis*, nisin A, thermized cheese milk

## Abstract

The capacity of growth, survival, and adaptive responses of an artificial contamination of a three-strain *L. monocytogenes* cocktail in factory-scale thermized (65 °C, 30 s) Graviera cheese milk (TGCM) was evaluated. Bulk TGCM samples for inoculation were sequentially taken from the cheese making vat before process initiation (CN-LM) and after addition of a commercial starter culture (CSC), the CSC plus the nisin A-producing (NisA+) costarter strain *Lactococcus lactis* subsp. *cremoris* M78 (CSC + M78), and all ingredients with the rennet last (CSC + M78-RT). Additional treatments included *Listeria*-inoculated TGCM samples coinoculated with the NisA+ costarter strain M78 in the absence of the CSC or with the CSC in previously sterilized TGCM to inactivate the background microbiota (CSC-SM). All cultures were incubated at 37 to 42 °C for 6 h, followed by additional 66 h at 22 °C, and 48 h at 12 °C after addition of 2% edible salt. *L. monocytogenes* failed to grow and declined in all CSC-inoculated treatments after 24 h. In contrast, the pathogen increased by 3.34 and 1.46 log units in the CN-LM and the CSC-SM treatments, respectively, indicating that the background microbiota or the CSC alone failed to suppress it, but they did so synergistically. Supplementation of the CSC with the NisA+ strain M78 did not deliver additional antilisterial effects, because the CSC *Streptococcus thermophilus* reduced the growth prevalence rates and counteracted the *in situ* NisA+ activity of the costarter. In the absence of the CSC, however, strain M78 predominated and caused the strongest *in situ* nisin-A mediated effects, which resulted in the highest listerial inactivation rates after 24 to 72 h at 22 °C. In all TGCM treatments, however, *L. monocytogenes* displayed a “tailing” survival (1.63 to 1.96 log CFU/mL), confirming that this pathogen is exceptionally tolerant to cheese-related stresses, and thus, can't be easily eliminated.

## 1. Introduction

*Listeria monocytogenes* is a foodborne pathogen of great concern and a serious microbial hazard for the dairy industry, and it has been detected in various retail cheeses made of raw or pasteurized milk [1]–[3]. The public health concerns associated with its presence in cheese have been demonstrated by several recent listeriosis outbreaks, mostly linked to soft cheeses [4]–[6]. Therefore, many product-specific validation studies conducted to assess the growth potential of *L. monocytogenes* during processing and storage of various retail cheeses have been critically reviewed in recent years [7],[8]. Most of them were particularly required in Greece and other EU-member countries in response to the microbiological safety criteria in Regulation (EC) 2073/2005 and its amended Regulation (EC) 1441/2007 which specify a maximum allowable *L. monocytogenes* level of 100 CFU/g or ml in ready-to-eat (RTE) foods which do not support growth of the pathogen (i.e., foods with pH ≤ 4.4, or a_w_ ≤ 0.92, or pH ≤ 5.0 and a_w_ ≤ 0.94, or a shelf life < 5 days) [9],[10].

Although most traditional cooked-hard, hard or semi-hard cheeses, such as the Greek Graviera [11],[12], the French Cantal [13], and the popular varieties Edam, Gouda [14],[15] and Cheddar [16],[17] usually have pH and a_w_ values higher than the EC regulatory “safe-value” ranges, they are not supportive of *L. monocytogenes* growth [11]–[17]. Hence, all the above RTE cheese products will not exceed the limit of 100 CFU/g throughout their commercial shelf life, but they may harbor fewer than 100 viable cells because *L. monocytogenes* has high adaptive stress tolerance responses [18],[19]. Especially the low or moderate levels of acid and saline stresses encountered in the core or the surface of hard and semi-hard cheese [16],[20],[21] may result in tailing survival patterns during ripening and storage [11],[15],[22],[23]. However, survival of *L. monocytogenes* is not desired in any RTE food. Therefore, general food safety recommendations emphasize the need for implementation of effective control measures so that RTE cheese products are free of this deadly pathogen [1].

One of the most promising measures to enhance inactivation of *L. monocytogenes* in cheese may be the addition to the processed milk of novel bacteriocin-producing (Bac+) strains of lactic acid bacteria (LAB) as starter, costarter or adjunct bioprotective cultures [24]–[26]. Wild Bac+ *Lactococcus lactis* strains are probably the best candidates for inhibiting *L. monocytogenes* in various cheeses [27]–[30]. *L. lactis* strains that produce nisin A (NisA+), nisin Z or lacticins 3147 and 481, singly or in combinations, have been evaluated as antagonistic costarter strains, supplementing the primary starter culture, in cheese and other dairy products [25],[31]–[33]. Overall, the direct use of Bac+ (Nis+) starter or adjunct cultures in fermented dairy products appears to be more advantageous than addition of nisin (e.g., Nisaplin) or other bacteriocins because it may solve several problems associated with the *in situ* loss of activity or degradation of the active peptides by irreversible binding on milk fat globules, enzymatic inactivation or other detrimental factors [25],[31],[34]. Encapsulation may offer an alternative option for extending nisin activity during ripening, as shown in Cheddar cheese [35]. Also, because nisin is more active at low pH, major (3–5 log unit reductions) of *L. monocytogenes* or *L. innocua* have been reported after direct addition of nisin or Nis+ adjunct strains in fermented cheese products of pH 4.0–5.0, such as cottage cheese [29],[36]–[38]. Conversely, limited to negligible antilisterial effects of nisin or Nis+ adjunct strains have been reported in ripened cheeses of pH > 5.0, or when the nisin concentrations were reduced, or nisin-hindering factors or resistant mutants of *L. monocytogenes* were present [39]–[42]. Combining Nis+ *L. lactis* with multi-strain commercial starter cultures requires careful evaluations because the nisin producer may become detrimental by reducing performance of the starter, or reversibly, it may become beneficial by increasing starter lysis and thus the release of intracellular enzymes during ripening [25],[43].

Recent studies of the Dairy Research Institute focused on the commercial application of an indigenous NisA+ *L. lactis* subsp. *cremoris* rare genotype (strains M78 and M104) isolated from Greek raw milk [44],[45] as novel, craft-made, costarter adjunct culture in traditional Greek cooked hard cheese manufacture [46]. With regard to the NisA+ activity of strains M78 and/or M104, it was strong in pure culture in model sterile cheese milk [47] as well as in commercially thermized milks [48]. Both strains, however, failed to exhibit similar strong NisA+ activity levels in thermized Graviera cheese milks (TGCM) and model Graviera minicheeses manufactured with addition of CSCs containing *Streptococcus thermophilus* as the primary cheese acidifier [46],[49]. These findings prompted to conduct quantitative real-time PCR (qRT-PCR) studies, which revealed that the NisA+ costarter strain M78 was capable of promoting sufficient growth and *nisA* gene expression in the core of commercial CSC-inoculated Graviera cheeses (14–15 kg each) for at least one week after manufacture [50]. Both *nisA* gene expression and *in situ* residual nisin A activity were, however, reduced to minimization during ripening, despite the producer strain M78 remained viable at fairly high growth prevalence rates in the ripened cheese matrix and retained its direct NisA+ activity *in vitro* [50]. Several interacting hurdle factors appeared to be responsible for the observed reductions in *nisA* gene expression and residual nisin A activity in commercial Graviera cheese products [50].

Therefore, this challenge study was conducted in direct correspondence with the above qRT-PCR Graviera cheese study of Noutsopoulos et al. [50] to specify main antagonistic effects. Overall, we showed that the primary hurdle factors which appeared to reduce the growth and *in situ* nisin A production and activity of this novel *L. lactis* subsp. *cremoris* costarter culture were responsible for the simultaneous growth suppression of the artificial *L. monocytogenes* contamination in factory-scale prepared TGCMs and in simulating Graviera cooked cheese curd environments. Practical aspects to overcome the above ecological and technological controversies are also discussed.

## 2. Materials and methods

### 2.1. Listeria monocytogenes strains and preparation of listerial inocula

Two virulent *L. monocytogenes* strains of cheese origin, ISS G79 (serotype 1/2b) and ISS G185 (serotype 1/2a) plus *L. monocytogenes* no.10 (serotype 4ab), an avirulent reference strain, were used in accordance with previous studies [44],[46],[49]. Frozen (−30 °C) stock cultures of each strain in tryptic soy broth with 0.6% yeast extract (Lab M, Heywood, UK) plus 20% glycerol were activated twice (30 °C, 24 h) in 10 mL of brain heart infusion (BHI) broth (Lab M) before use. For inoculation purposes, fresh (24-h) 10-mL BHI cultures of the strains were centrifuged (3,200 × *g* for 15 min), the cells were washed with 10 mL of quarter-strength Ringer solution (Lab M), resuspended in the same diluent, and suspensions were combined as before [11],[44],[51]. Afterward the three-strain cocktail of *L. monocytogenes* was diluted to yield an inoculum in milk of approximately 10^3^ CFU/mL [48].

### 2.2. Starter and costarter LAB strain preparations

*Lactococcus lactis* subsp. *cremoris* M78 was selected as a craft-made NisA+ costarter strain in correspondence with previous qRT-PCR studies [47],[50]. All antilisterial treatments were done in bulk TGCM (65 °C, 30 s) portions used to produce the Graviera cheese samples analyzed by Noutsopoulos et al. [50]. The cheeses were prepared in a local semi-industrial plant (Skarfi E.P.E.-Pappas Bros. Traditional Dairy, Filippiada, Greece). The CSC added to TGCM bulks was a freeze-dried concentrated blend of *L. lactis* subsp. *lactis*, *L. lactis* subsp. *cremoris*, *L. lactis* subsp. *lactis* biovar *diacetylactis, Leuconostoc mesenteroides* subsp. *cremoris* and *Streptococcus thermophilus* strains (GRU IDC 01, Centro Sperimentale del Latte [CSL], Lodi, Italy). One packaging size (recommended inoculation 4 doses per 2,000 L of milk) of this Direct Vat Set (DVS) CSC was added to the cheese vat; the mixed CSC inoculation level was ca. 6 log CFU/mL of milk. Strain M78 was activated twice (30 °C, 24 h) in 10 mL of MRS broth (Lab M) and was subcultured in sterile (121 °C, 5 min) 10% reconstituted skim milk (RSM) (Lab M) for direct vat inoculation (ca. 6 log CFU/mL also) of the TGCM in the plant [50] and parallel use in the present challenge experiments.

### 2.3. Cheese milk samples

Bulk raw 90% ewes' and 10% goats' milk mixtures (two individual batches; 2 to 3 tones each) collected from local farmers were thermized and then cooled at 34 °C before starter LAB inoculation followed by renneting, curdling and curd cutting operations. Each bulk TGCM batch was used for the manufacture of two Graviera cheese vats (1,000 L each), one with the aforementioned CSC added only and the other with the CSC plus strain M78 (CSC + M78) [50]. In this study, the capacity of the above three-strain cocktail of *L. monocytogenes* to grow and/or survive in TGCM portions withdrawn from the CSC + M78 cheese vat before and during the above early cheese processing operations was assessed. Aseptically collected samples (100 mL) from each TGCM bulk before initiation of cheese processing were transferred to the DRI microbiology laboratory at Ioannina in insulated ice boxes, and analyzed microbiologically and for pH as described by Samelis et al. [44].

### 2.4. Factory-scale prepared TGCM and fresh Graviera cheese curd challenge experiments

Because artificial contamination of the bulk TGCM with *L. monocytogenes* was prohibited at the commercial plant level [50], an alternate experimental approach was followed. Six antilisterial challenge treatments were set in duplicate, using 12 presterilized 500-mL Duran flasks poured with 200-mL each of bulk TGCM before and during Graviera cheese manufacture (Table 1). To avoid postthermal contaminations, the milk samples of three out of the six challenge treatments serving as different controls in the experiments, CN-LM, M78 and CSC-SM, were aseptically collected from the edge of the stainless steel pump pouring the bulk TGCM into the curdling vessel [50]. Until collection of all later TGCM samples in Table 1, the four flasks of the CN-LM and M78 treatments were tempered in a water bath at 34 °C, while the two flasks used for the CSC-SM treatment were heat-sterilized (121 °C; 5 min) and then tempered at 34 °C. Meantime, after pouring 1,000 L of bulk TGCM in the CSC + M78 cheese vat under continuous stirring, 200-mL milk sample portions for the remaining CSC, CSC + M78 and CSC + M78-RT treatments were collected by a flamed milk sampler directly from the cheese vat (Table 1). Particularly for the CSC + M78-RT treatment, the rennet was added last, after addition of all other ingredients to the cheese milk, which then was left to curdle at 34 °C. Concurrently, the six pre-tempered flasks of the three different control treatments, M78, CN-LM, and CSC-SM, were prepared (Table 1). Particularly for the CSC-SM model treatment, the flasks were inoculated with 0.2 mL each of 0.2 g CSC powder diluted in 10 mL of 10% RSM to yield ca. 6 log CFU/mL of mixed starter LAB strain populations against the pathogen, as in the corresponding CSC treatment, which contained the background microbiota and enzymes with potent inhibitory activity present in TGCM. Afterward all flasks were inoculated with ca. 3 log CFU/mL of the *L. monocytogenes* cocktail (Table 1) and incubated at 37 °C for initial 2 h. During this period, only the two RT flasks developed a firm curd, which was simulative of the CSC + M78 cheese curd before its cutting [50]. The CSC + M78-RT curds were broken by vigorous shaking of the flasks, and incubation of all flasks was continued at 42 °C for 1 h in simulation of the cheese curd cooking process. The flasks were then returned in the 37 °C incubation chamber for another 3 h and then were shifted at 22 °C in a cooling incubator for additional 66 h. The excessive whey that was separated above the broken granular curd in the CSC + M78-RT flasks only was not drained out to maintain equal cultured milk volumes in all challenge treatments. After 72 h, 2% edible sea salt was added to each flask and incubation continued at 12 °C for 5 days (120 h) to simulate the brining process at the usual salt level of ripened Graviera cheeses [20],[46]. All samples were analyzed at inoculation (0 h), after 3 h at 37 to 42 °C, another 3 h at 37 °C, 24 and 72 h at 22 °C, and 120 h at 12 °C.

### 2.5. Analysis of cultured milk and fresh curd samples

At each sampling time, each TGCM or fresh curd flask was agitated by hand and 1-mL of cultured milk was transferred in tubes with 9-mL of sterile Ringer solution. Particularly for the RT cultures which were difficult to pipette, 5 g samples were homogenized with 45 mL of Ringer solution in a stomacher bag (Lab Blender, Seward 400, London, UK) for 2 min to obtain the first decimal dilution. Then appropriate dilutions of each sample in Ringer solution were plated in duplicate on total and selective agar media. *L. monocytogenes* was enumerated on Palcam agar (Lab M) incubated at 30 °C for 72 h. Separate enumerations of mesophilic and thermophilic LAB were made on duplicate series of M17 agar (Lab M) plates incubated at 22 °C for 72 h or 42 °C for 48 h, respectively. Indigenous enterococci were selectively enumerated on Slanetz & Bartley agar (Lab M), incubated at 37 °C for 48 h, whereas total staphylococci were enumerated on Baird-Parker agar with egg yolk tellurite (Lab M), incubated at 37 °C for 48 h.

**Table 1.** Challenge treatments against *Listeria monocytogenes* conducted during processing of commercially thermized Graviera cheese milk inoculated with a CSC^1^ and/or the novel, nisin A-producing costarter strain *Lactococcus lactis* subsp. *cremoris* M78^2^.

Challenge treatment code	Type of cheese milk	Cheese milk collection step	Presence of background microbiota	CSC inoculation level (log CFU/mL)	Strain M78 inoculation level (log CFU/mL)	Presence of rennet	Artificial listerial contamination level (log CFU/mL)
CN-LM	Thermized (65 °C; 30 sec)	After thermization	Yes	N/A^3^	N/A	No	3.0
M78	Thermized (65 °C; 30 sec)	After thermization	Yes	N/A	6.0	No	3.0
CSC	Thermized (65 °C; 30 sec)	After inoculation with the CSC	Yes	6.0	N/A	No	3.0
CSC + M78	Thermized (65 °C; 30 sec)	After inoculation with strain M78 as costarter	Yes	6.0	6.0	No	3.0
CSC + M78-RT	Thermized (65 °C; 30 sec)	After renneting and setting of the milk to curdle	Yes	6.0	6.0	Yes	3.0
CSC-SM	Sterilized (121 °C; 5 min)	Postthermization followed by heat sterilization	No	6.0	N/A	No	3.0

^1^CSC: Commercial starter culture.

^2^Two 500-mL Duran flasks containing 200 mL milk samples each were analyzed per treatment. The milk in all flasks was tempered at 34 °C, either in a water bath or into the double-jacketed cheese curdling vessel, before and during the sample preparation procedures. All challenge treatments were replicated twice by collecting milk samples from two individual Graviera cheese productions; viz. Noutsopoulos et al. [50].

^3^N/A, not applied.

Especially for the CSC-SM cultures, samples were plated on M17 agar plates incubated at 45 °C for 48 h (selective for enumerating CSC *S. thermophilus*; non-supportive of CSC *L. lactis* growth) and on MRS agar plates (Lab M) incubated at 22 °C for 72 h (selective for enumerating all CSC *L. lactis*; non-supportive of CSC *S. thermophilus* growth) [50]. The pH of all cultures was measured by a Jenway 3510 digital pH meter (Essex, UK) equipped with a glass electrode.

### 2.6. Selective enumerations of the NisA+ colonies of the costarter strain M78

After total colony enumerations, selective enumerations of the NisA+ colonies of strain M78 were performed on the first series of M17 plates at 22 and 42 °C at 6 and 72 h of incubation by the agar overlay assay, as described [49] and illustrated [46] previously. The avirulent *L. monocytogenes* strain no.10 was the indicator; the M17 agar plates of milk cultures without strain M78 inoculation served as controls. The second series of M17 agar plates was overlayed with lawns of strain M78 seeded in melted (45 °C) M17 agar to detect its potential inhibition by indigenous Bac+ LAB strains and assure its immunity to nisin A. Occasionally, for confirmatory purposes, the 6-h and 72-h TGCM samples were also plated on milk plate count agar (MPCA; Lab M) incubated at 37 °C and 45 °C for 48 h. Afterward the MPCA agar plates were also overlayed with *L. monocytogenes* no.10 to verify the growth prevalence rates of strain M78 at 37 °C as compared to the M17 agar plates at 22 °C and 42 °C, and to ensure absence of NisA+ colonies at 45 °C, where strain M78 is unable to grow [45].

### 2.7. In situ detection of nisin A activity in cultured cheese milks and curds

The well diffusion assay of Lianou and Samelis [48] was applied for *in situ* detection of nisin A activity in all TGCM cultures and the CSC-SM cultures after 6 and 72 h of incubation. Strain no.10 was again the indicator. Because the clotted milk samples could not be filter-sterilized, they were filled in the 6-mm diameter wells after their pH was adjusted at 5.8 with 1 N NaOH (Merck, Darmstadt, Germany) and then heated at 80 °C for 15 min in a water bath. Acidic (unadjusted) heated samples and the original unheated samples containing the total viable cells of each milk coculture were evaluated comparatively by filling duplicate wells for each of the three occasions (acid/viable; acid/heated; neutralized/heated) of each milk sample on the same plate. After filling the wells, all TSAYE plates were kept at 4 °C for 2 h for sample diffusion and then incubated at 30 °C overnight to develop inhibition zones. The net diameter of inhibition zones (total diameter of the “external” zone in X mm minus 6 mm occupied by the “internal” diameter of each well on the plates) was measured with a micrometer (Mitutoyo, model D15, Kanagawa, Japan).

### 2.8. Statistical analysis

All experiments of this study were replicated twice by analyzing two individual samples per replicate (n = 4). The microbiological data were converted to log_10_ units and along with the pH data were subjected to a one-way analysis of variance using the Statgraphics Plus for Windows Version 5.2 (Manugistics, Inc., Rockville, MD) software. The means were separated by the least significance difference (LSD) procedure at the 95% confidence level (*P* < 0.05) for determining the significance of differences in each treatment with time and between different treatments on each sampling day.

## 3. Results and discussion

### 3.1. Behaviour of L. monocytogenes in TGCM and fresh curd challenge treatments with or without CSC and/or strain M78 inoculation

**Figure 1.**
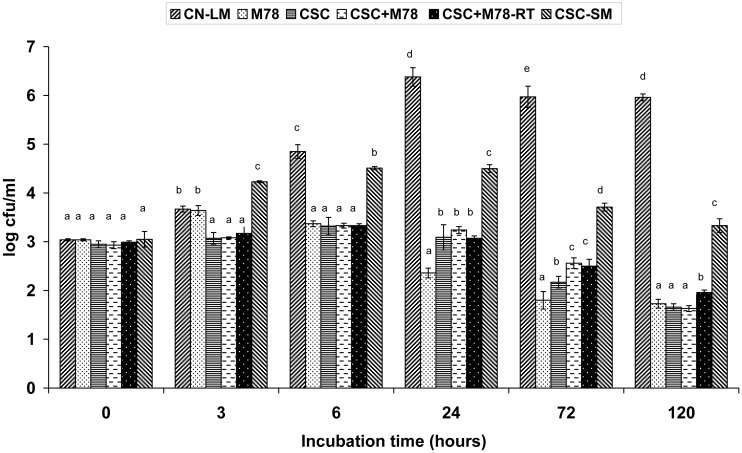
Populations (mean ± standard deviation; n = 4) of *Listeria monocytogenes* in artificially contaminated, factory-scale thermized Graviera cheese milk (TGCM) samples manufactured with addition of a commercial starter culture (CSC) or the CSC combined with the nisin A-producing (NisA+) *Lactococcus lactis* subsp. *cremoris* M78 strain before (CSC + M78) or after (CSC + M78-RT) the rennet was added. Additional challenge treatments included artificially contaminated TGCM samples without CSC or M78 strains' inoculation (CN-LM) or coinoculated with the pathogen and the NisA+ strain M78 in the absence of the CSC (M78), or coinoculated with the CSC only in previously heat-sterilized milk samples (CSC-SM). Means corresponding to the same incubation time interval and lacking a common letter are significantly different (*P* < 0.05). Data for 48 h of incubation are not shown for graph simplification.

*L. monocytogenes* failed to grow (*P* > 0.05) in the presence of the CSC in TGCM. Specifically, the cocktail grew slightly (≤0.4 log units) in all the CSC-inoculated TGCM samples within the first 6 h, while its viable populations declined significantly after 72 h, and continued to decline in the salted (2%) cultures after 120 h (Figure 1). Supplementation of the CSC with the NisA+ costarter strain in TGCM (CSC + M78) and curd (CSC + M78-RT) did not enhance pathogen inactivation; instead *L. monocytogenes* survived better (*P* < 0.05) compared with the control CSC treatment after 72 h at 22 °C (Figure 1). In agreement, previous supplementation of a similar CSC with strains M78 and M104 did not reduce the survival of an artificial 3-log listerial contamination in comparison with the CSC *per se* during processing and storage of model Graviera minicheeses [46]. In that previous study, however, an avirulent *Listeria* spp. cocktail, which included strain no.10, was inoculated in the finished curd postcooking [46]. Therefore, this study was suitably designed to evaluate the growth potential of listerial contamination during TGCM curdling and curd cooking at 34 to 42 °C (Table 1). The results in Figure 1 assured that this particular CSC minimized growth of ca. 1000 cells/mL of *L. monocytogenes* during early Graviera cheese processing steps that are at the highest risk probability as regards growth of natural *L. monocytogenes* contamination in hard cheese [12],[38],[52].

The behaviour of *L. monocytogenes* in TGCM with strain M78, but without the CSC, was quite similar with that in the CSC-containing TGCM cultures (Figure 1). Two significant differences were: (i) growth initiation of the pathogen within the first 3-h period at 37 to 42 °C; and (ii) overall greater declines of the pathogen's viability after 24 to 72 h at 22 °C followed by a more “tailing” survival in the salted M78 cultures compared with the salted CSC-containing TGCM cultures at 12 °C after 120 h. Meantime, in contrast with all LAB-inoculated challenge treatments, *L. monocytogenes* promoted major growth and exceeded the 6-log population level in TGCM without LAB inoculation at 24 h (Figure 1). Thus, at first glance, the total natural antilisterial hurdle effect of TGCM seemed very weak and a meaningless contributor to the inhibitory effects of strain M78 and/or the CSC against *L. monocytogenes*, especially during the first 6 h of incubation at 37 to 42 °C (Figure 1). To our great surprise, however, when the background microbiota and indigenous enzymes with potent inhibitory activity in TGCM, such as peroxidase, were inactivated by heat sterilization, the CSC *per se* failed to control growth of *L. monocytogenes* in the model sterile milk (CSC-SM) to the same extent it did in the factory-scale CSC-inoculated TGCM (Figure 1). Hence, the indigenous microbiota and possibly inhibitory enzymes remaining active in TGCM contributed to increase the antilisterial effects of the CSC. Conversely, their corresponding net effects in isolation, i.e., in the absence of the CSC in the LM-CN treatment, were the most delayed and weakest among all TGCM treatments (Figure 1).

### 3.2. Changes in the cultured milk pH during incubation of TGCM and fresh curd challenge treatments with or without previous CSC and/or strain M78 inoculation

**Figure 2.**
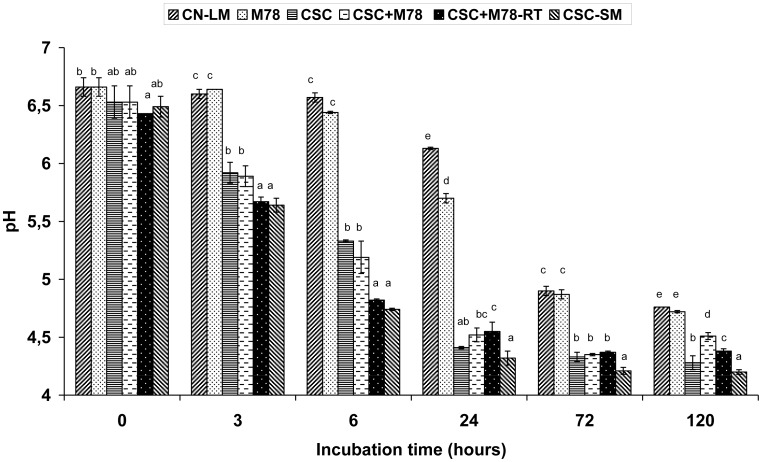
Values of pH (mean ± standard deviation; n = 4) of artificially *Listeria*-contaminated, factory-scale thermized Graviera cheese milk (TGCM) samples manufactured with addition of a commercial starter culture (CSC) or the CSC combined with the nisin A-producing (NisA+) *Lactococcus lactis* subsp. *cremoris* M78 before (CSC + M78M) or after (CSC + M78-RT) the rennet was added. Additional challenge treatments included artificially contaminated TGCM samples without CSC or strain M78 inoculation (CN-LM) or coinoculated with the pathogen and the NisA+ strain M78 in the absence of the CSC (M78), or coinoculated with the CSC only in previously heat-sterilized milk samples (CSC-SM). Means corresponding to the same incubation time interval and lacking a common letter are significantly different (*P* < 0.05). Data for 48 h of incubation are not shown for graph simplification.

The pH drop was faster (*P* < 0.05) in all CSC-inoculated TGCM cultures than in the TGCM control and M78 cultures (Figure 2), reflecting superior milk acidifying capacities of the CSC particularly of *S. thermophilus* during the first 6 h of incubation at 37–42 °C. A major early drop of the mean pH from 6.4 after cooking (day-0) to 5.4 after 24 h (day-1) of fermentation also occurred in the corresponding CSC and CSC + M78 Graviera cheeses; following this reduction, however, the cheese pH did not change significantly during brining and ripening for 30 days [50]. Hence, the extent of pH drop (final pH 4.28–4.51) in all CSC-containing TGCM treatments (Figure 2) was greater than the final pH in the core of the corresponding RTE Graviera cheeses (14–15 kg each) by 0.9 to 1.1 pH units [50]. Meanwhile, the final pH values of the TGCM control cultures without CSC inoculation (LM-CN and M78) remained the highest (4.72–4.76), whereas the lowest cultured milk pH of 4.2 occurred in the absence of the background microbiota in the CSC-SM cultures (Figure 2).

The above great differences in pH were because during Graviera cheese manufacture most of the whey is removed after curd cutting and cooking, and thus, the moisture content and a_w_ of the fresh curds are reduced after cheese pressing. On the contrary, in this study, the cheese whey was on purpose retained into the TGCM flasks. This apparently increased the availability of lactose and other carbohydrates fermented by the CSC, costarter M78 and indigenous LAB. Consequently, the milk pH fell much below 5.0 during incubation (Figure 2). Moreover, conditions in all TGCM cultures were moister than in the core of fresh Graviera cheese moulds [50]. Particularly as regards their low pH values, the CSC-inoculated TGCM cultures resembled with typical soft acid-curd cheeses, such as cottage cheese or the Greek PDO Galotyri cheeses [29],[53]. Previous sterilization of TGCM did not reduce but rather enhance acidification by the CSC in the absence of other LAB in CSC-SM (Figure 2). Because the survival of *L. monocytogenes* was 100-fold higher (*P* < 0.05) in the CSC-SM cultures than in the LAB-inoculated TGCM cultures (Figure 1), it was evident that the greater pathogen declines in the model SM cultures were not solely due to acidification (Figure 2).

### 3.3. Behaviour of the technological LAB microbiota in TGCM and fresh curd challenge treatments with or without CSC and/or strain M78 inoculation

The bulk TGCM samples had the following total and LAB populations (mean log CFU/mL ± standard deviation): 4.60 ± 0.12 (MPCA/37 °C), 4.03 ± 0.11 (M17/22 °C), 3.45 ± 0.21 (M17/42 °C), 3.15 ± 0.21 (MRS/30 °C), 2.72 ± 0.33 (MRS/45 °C). Spontaneous enterococci, staphylococci and coliforms were 2.84 ± 0.08, 1.15 ± 0.21 and 0.73 ± 0.18 log CFU/mL, respectively, whereas yeasts were absent/mL of TGCM (data not tabulated). Due to the higher (65 °C) thermization temperature of the present bulk milks, their above populations were lower by 1.0 to 1.5 log units compared with those of bulk milks previously thermized at 63 °C for 30 s [49]. Similar observations were made when the same raw bulk milk batches were thermized comparatively at 60 °C or 67 °C for 30 s [44].

During the first 6 h, total LAB populations exceeded the 8-log level in all treatments, except in the uninoculated TGCM control (Table 2). Growth of the CSC *S. thermophilus* on M17 agar plates at 42 °C was more pronounced (*P* < 0.05) than that of the CSC *L. lactis* and other mesophilic LAB on M17 agar plates at 22 °C (Table 2), as also it was during fermentation of the corresponding Graviera cheese batches [50]. Compared with the real, factory-scale prepared CSC + M78 cheeses, however, the prevalence (%) of the NisA+ costarter strain was remarkably lowered in the CSC + M78 and CSC + M78-RT cultures after 6 h and decreased further after 72 h of incubation (Table 2). Specifically, on the M17 agar plates at 22 °C, which were selective for *L. lactis* strains, growth prevalence rates of strain M78 ranged from 12.0 to 52.5% (Table 2), while its corresponding rates in the CSC + M78 cheeses were as high as 91.2 to 100% [50]. On the least selective for *L. lactis* M17 agar plates at 42 °C, the prevalence rates of strain M78 in CSC + M78 and CSC + M78-RT cultures declined at very low levels (<0.4 to 3.4% in Table 2) compared to its corresponding rates (19.9 to 38.9%) in the CSC + M78 cheeses [50].

The failure of strain M78 to predominate in the CSC-containing TGCM cocultures (Table 2) was attributed to the harsher acid/low pH conditions created by the CSC *S. thermophilus* upon its growth as the primary acidifier than its absence (M78) from the TGCM environment (Figure 2). The early predominance and strong acidification of *S. thermophilus* also had prominent adverse effects on the growth prevalence rates of the CSC *L. lactis* strains in the model CSC-SM cultures (Table 3). Indeed the viability of the CSC *L. lactis* declined dramatically in the salted (2%) cultures at 12 °C (Table 3) because of the acid stress, or unless those CSC *L. lactis* strains also possessed genetically encoded autolytic properties for accelerating cheese ripening [43],[54]. The later hypothesis may be valid because the CSC *L. lactis* also declined greatly in the ripened CSC + M78 cheeses of pH 5.2 to 5.6, unlike the wild NisA+ strain M78 which retained fairly high (ca. 25%) prevalence rates [50]. No CSC *L. mesenteroides* subsp. *cremoris* were detected in the present cultures (Tables 2 and 3) and the corresponding Graviera cheeses [50]. This gas-producing starter type was also undetectable in the freeze-dried CSC powder analyzed for isolation of its primary LAB constituents, according to Vandera et al. [51].

**Table 2.** Populations (log CFU/mL; mean ± standard deviation, n = 4) of total lactic acid bacteria and corresponding populations and growth prevalence rates (%) of the nisin A-producing (NisA+) *Lactococcus lactis* subsp. *cremoris* M78 strain in *Listeria*-inoculated, commercially TGCM^1^ samples, as determined by the agar overlay method.

Incubation time (hours)	Challenge treatment^2^	Rennet	Populations of lactic acid bacteria (LAB) enumerated on						

			M17 agar at 22 °C			M17 agar at 42 °C			S&B agar at 37 °C

			Total LAB count	Strain M78 count	Prevalence (%) ^3^	Total LAB count	Strain M78 count	Prevalence (%)^3^	*Enterococcus* counts
6	CN-LM	No	5.42 ± 0.09 a	N/A	N/A	5.05 ± 0.19 a	N/A	N/A	4.24 ± 0.10 b
	M78	No	8.30 ± 0.29 ef	8.30 ± 0.29 ef	100	8.47 ± 0.10 ef	8.47 ± 0.10 ef	100	2.93 ± 0.34 a
	CSC	No	8.33 ± 0.07 e	N/A	N/A	8.81 ± 0.09 f	N/A	N/A	5.88 ± 0.06 e
	CSC + M78	No	8.34 ± 0.08 e	8.06 ± 0.03 d	52.5	8.86 ± 0.09 f	7.39 ± 0.55 cd	3.4	5.49 ± 0.11 de
	CSC + M78-RT	Yes	8.30 ± 0.09 e	7.84 ± 0.34 d	34.7	8.76 ± 0.11 f	<7.00 b	<1.7	5.34 ± 0.08 d
72	CN-LM	No	8.91 ± 0.41 f	N/A	N/A	8.64 ± 0.13 ef	N/A	N/A	8.49 ± 0.23 f
	M78	No	8.89 ± 0.21 f	8.89 ± 0.21 f	100	8.80 ± 0.04 f	8.80 ± 0.04 f	100	4.80 ± 0.07 c
	CSC	No	8.83 ± 0.04 f	N/A	N/A	8.84 ± 0.07 f	N/A	N/A	5.98 ± 0.06 e
	CSC + M78	No	8.48 ± 0.15 ef	7.91 ± 0.43 d	26.9	8.84 ± 0.14 f	6.39 ± 0.13 b	0.4	5.74 ± 0.19 e
	CSC + M78-RT	Yes	8.11 ± 0.10 d	7.19 ± 0.26 c	12.0	8.40 ± 0.42 ef	<6.00 a	<0.4	5.71 ± 0.13 e

^a–f^: For each enumeration agar medium, means within a column and row lacking a common letter are significantly different (*P* < 0.05).

^1^TGCM: Thermized Graviera cheese milk.

^2^Treatments: CN-LM, *Listeria*-inoculated TGCM with no further inoculation; M78, TGCM inoculated with the NisA+ strain M78; CSC, TGCM after addition of the commercial starter culture; CSC + M78, TGCM after addition of the costarter NisA+ strain M78; CSC + M78-RT, TGCM after addition of all other cheese making ingredients with the rennet last.

^3^Prevalence (%): Mean colony population of the NisA+ strain M78 × 100 divided by the mean population of total LAB colonies counted on the plates.

**Table 3.** Populations (log CFU/mL; mean ± standard deviation, n = 4) and growth prevalence rates (%) of the CSC^1^ strains in heat-sterilized cheese milk, coinoculated with *Listeria monocytogenes*, during incubation for 120 h.

Starter LAB species included in the CSC preparation	Incubation conditions							
	Inoculation (0 h)		After 6 h at 37 to 42 °C		After 72 h at 22 °C		After 120 h at 12 °C	

	Starter LAB population	Prevalence (%)^2^	Starter LAB population	Prevalence (%)^2^	Starter LAB population	Prevalence (%)^2^	Starter LAB population	Prevalence (%)^2^
*Streptococcus thermophilus*	6.24 ± 0.06 a	66.6	9.22 ± 0.08 d	84.3	9.28 ± 0.06 d	87.4	9.15 ± 0.06 d	99.4
Mixed mesophilic starter LAB strains of: *Lactococcus lactis* subsp. *lactis Lactococcus lactis* subsp. *cremoris* *Lc. lactis lactis* var. diacetylactis	5.94 ± 0.02 a	33.4	8.49 ± 0.03 c	15.7	8.44 ± 0.05 c	12.6	6.91 ± 0.04 b	0.6
*Leuconostoc mesenteroides* subsp. *cremoris*	Not detected	N/A	Not detected	N/A	Not detected	N/A	Not detected	N/A

^a–d^: Means within a column and row lacking a common letter are significantly different (*P* < 0.05).

^1^CSC: Commercial starter culture.

^2^Prevalence (%): Mean populations of the thermophilic (*Streptococcus thermophilus*) or the mesophilic (mixed *Lactococcus lactis* strains) portion of the CSC × 100 divided by the total CSC population (sum of thermophilic and mesophilic portion) enumerated in each heat-sterilized cheese milk sample.

**Table 4.** Well diffusion assay-based detection of *in situ* nisin A-mediated (NisA+) antilisterial activity by *Lactococcus lactis* subsp. *cremoris* M78 plus other type of bacteriocin-like (Bac+) antilisterial activity by the indigenous microbiota in thermized Graviera cheese milk (TGCM) samples coinoculated with *Listeria monocytogenes* after 6 and 72 h of incubation.

Inoculation treatment^1^	Replicate experiment	Incubation conditions					
		6 h at 37 to 42 °C			72 h at 22 °C		
		Acid/viable^2^	Acid/heated^3^	pH adjusted/heated^4^	Acid/viable	Acid/heated	pH adjusted/heated
CN-LM	1	−	−	N/A	++ 5.5/5.3	−	−
	2	−	−	N/A	++ 5.2/5.0	−	−
M78	1	++ 5.8/6.2	−	N/A	++ 7.0/7.1	++ 4.0/3.8	++ 3.0/2.9
	2	++ 6.0/6.2	−	N/A	++ 6.8/7.0	++ 4.2/4.0	++ 3.2/3.1
CSC	1	−	−	−	−	−	−
	2	−	−	−	−	−	−
CSC + M78	1	++ 3.0/4.0	−	−	−	−	−
	2	++ 3.0/4.0	−	−	−	−	−
CSC + M78−RT	1	+ 1.0/2.0	−	−	−	−	−
	2	+ 1.0/2.0	−	−	−	−	−
CSC−SM	1	−	−	−	(+)	−	−
	2	−	−	−	(+)	−	−

−, no inhibition zone; ++, very clear inhibition zone; +, clear inhibition zone; (+): faint and spread inhibition zone with non-defined edges due to acid; 5.5/5.3, numbers show the zone diameter (mm); N/A, not applied.

^1^Treatments: CN-LM, *Listeria*-inoculated TGCM with no further inoculation; M78, TGCM coinoculated with the NisA+ strain M78; CSC, TGCM after addition of the commercial starter culture; CSC + M78, TGCM after addition of the costarter NisA+ strain M78; CSC + M78-RT, TGCM after addition of all other ingredients with the rennet last; CSC-SM, the CSC was cocultured with *L. monocytogenes* in heat-sterilized (121 °C, 5 min) milk to inactivate the background microbiota and other natural inhibitors.

^2^Thermized (65 °C, 30 s) cultured milk samples were well-assay tested without any pretreatment in order to contain viable cells.

^3^Milk samples were heated at 80 °C for 15 min.

^4^Milk samples were heated at 80 °C for 15 min after adjustment of their pH at 5.8.

When, however, the CSC was absent in TGCM, the NisA+ strain M78 almost reached the 9-log level and total prevalence rates on the M17 agar plates, irrespective of incubation temperature (Table 2). Concomitantly, M78 cocultures retained strong inhibitory activities against *L. monocytogenes* no.10 in the well assays after the 72-h milk samples were heated at 80 °C (Table 4). In contrast, all 72-h milk samples of the CSC + M78 and CSC + M78-RT cultures, including the acid/viable samples, were negative, as also were the CSC and CSC-SM control samples at all incubation hours (Table 4). Hence the prevalence of viable M78 cells in the 72-h CSC-inoculated TGCM cultures had fallen so greatly (Table 2) that the costarter strain could not even exhibit direct NisA+ antagonistic activities against *L. monocytogenes* in the well assays (Table 4). Conversely, after 6 hours, the M78-containing cultures caused direct listerial inhibition by the viable NisA+ samples, which was stronger when the CSC was absent (Table 4). The corresponding heated (6-h) samples, however, were negative (Table 4) either because the nisin A molecules were still structured incompletely or because they were at insufficient amounts to inhibit *L. monocytogenes* in the absence of viable M78 cells in the wells.

**Figure 3.**
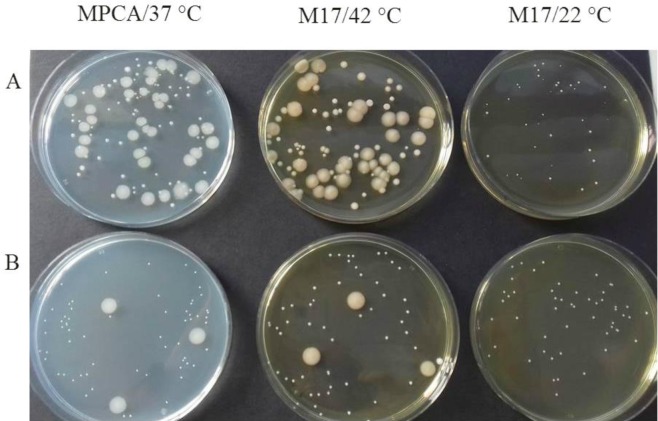
Predominant bacterial consortia grown on the highest dilution plates of milk plate count agar (MPCA) at 37 °C and M17 agar at 42 °C and 22 °C of thermized Graviera cheese milk (TGCM) samples without inoculation (A) or coinoculated with the nisin A-producing (NisA+) *Lactococcus lactis* subsp. *cremoris* M78 strain (B). Plates in row A show the indigenous TGCM microbiota, consisting of a great diversity of *Enterococcus* spp. colonies (whitish or creamy) of various colony sizes intermixed with a large glistering colony type of Gram-negative coliform bacteria capable for growth at 37 °C and 42 °C but not at 22 °C. Plates in row B show the very high predominance of the NisA+ strain M78 colonies (whitish and of same-size and appearance) on all three agar media and the ability of coliform bacteria to proliferate at approximately 10-fold lower levels on the plates in the presence of strain M78 under the same incubation conditions.

The TGCM cultures without CSC or M78 inoculation showed a very distinct pattern of microbial evolution during incubation (Table 2) accompanied by Bac+ antilisterial activities in all acid/viable milk samples at 72 h (Table 4). The reason was a predominant growth (30–60%) of spontaneous antagonistic *Enterococcus* colonies on M17 and MPCA agar plates (data not shown), which also grew at similarly high levels on SB agar plates at 72 h (Table 2). Spoilage gram-negative colonies of coliform bacteria also grew simultaneously at population levels as high as 8.41 ± 0.16 log CFU/mL in all 72-h TGCM control cultures. Their growth was abundant on M17 agar plates at 42 °C and MPCA plates at 37 °C, whereas they could not grow on M17 agar plates at 22 °C (Figure 3). Similar colonies of coliform bacteria grew at reduced levels of 7.75 ± 0.23 log CFU/mL on the respective agar plates of the control M78 cultures (Figure 3). Coliforms were unaffected by nisin A while the mild acidification capacity of the NisA+ strain M78 was insufficient to suppress their growth in TGCM (Figure 2). In contrast, coliforms were never detected in the CSC-containing TGCM cultures at 72 or earlier hours, irrespective of strain M78 presence, because they were suppressed below 4.0 log CFU/mL (data not shown) by the CSC, mainly *S. thermophilus* [55].

Addition of the CSC in TGCM also suppressed growth of enterococci by ca. 2.5 log units, which remained slightly below the 6-log level at 72-h. However, it was evident that enterococci managed major (3-log units) growth increases in all CSC-containing TGCM cultures during the first 6 hours of incubation at 37 to 42 °C (Table 2), as they also did in the corresponding Graviera cheeses [50]. The greatest growth suppression of enterococci in TGCM was noted in the control (plain) M78 cultures (Table 2). Suppression was probably due to the high prevalence (Table 2; Figure 3) and the increased *in situ* NisA+ activity (Table 4) rather than due to the mild acidification (Figure 2) of strain M78. Conversely, the predominant growth of the CSC, mainly *S. thermophilus*, in TGCM was against the subdominant costarter strain M78 to produce nisin A at levels sufficient to affect enterococcal growth in the CSC + M78 and CSC + M78-RT milks (Table 2). Similarly, strain M78 had no prominent NisA+ inhibitory effects against *Enterococcus* spp. in the corresponding CSC + M78 Graviera cheeses [50].

Staphylococci grew only in TGCM without LAB inoculation and reached 3.83 ± 0.53 log CFU/mL at 72 h. In contrast, staphylococci were undetectable (<10 CFU/mL) in TGCM inoculated with the CSC and/or strain M78 (data not tabulated). Elimination of staphylococci was also due to the acid/low pH stress imposed by the CSC (Figure 2). The *in situ* NisA+ activity (Table 4) might have contributed to staphylococcal elimination in the plain M78 cultures. On the contrary, staphylococci were not eliminated, but instead grew by ca. 2-log units in the corresponding CSC and CSC + M78 cheeses during fermentation and remained at 4–5 log CFU/g in the brined and ripened cheeses which, as mentioned, had a moderate pH of 5.2–5.6 [50].

### 3.4. Practical cooked hard cheese technology and safety aspects

The most important finding with regard to Greek Graviera cheese safety was the inability of *L. monocytogenes* to increase in factory-scale TGCMs and simulating curds. The prolific growth of the CSC *S. thermophilus* and the resultant acidification of the TGCMs and RT-curds within 3 to 24 h were the primary hurdle factors against growth of the pathogen, as it was also reported for Cantal cheese [13]. Additionally to the low pH (Figure 2), a part of the L-lactic acid formed by the CSC *S. thermophilus* and *L. lactis* strains would be of increased antilisterial activity in its undissociated form. Wemmenhove et al. [21] measured minimal inhibitory concentrations of undissociated lactic, acetic, citric and propionic acid for *L. monocytogenes* under conditions relevant to cheese. Most recently, they specified that growth of *L. monocytogenes* is not supported when the undissociated lactic acid concentration is >6.35 mM; this concentration is exceeded in the water phase of Gouda cheese when the total lactic acid content is >0.86% at a pH < 5.25 for fresh cheese or >1.26% at a pH < 5.50 for mature cheese [56]. In previous studies, we have also measured total organic acid concentrations in the core of fresh (day-1) or mature (day-23 to day-60) Graviera cheeses in association with their pH [20],[46],[57]. In all cases, lactic acid was the most abundant at total concentration levels ranging from 0.55% to 1.25% at a pH of 5.9 to 5.3, depending on the cheese age and mould size. In general agreement, growth of *L. monocytogenes* was not supported in the core [12] or on the cut-surface of ripened Graviera cheeses [11]. Another meaningful comparison is that levels of total lactic acid in fresh (24 h-old) model Graviera minicheeses (pH 5.7–5.9) and fresh RTE acid-curd Galotyri cheeses (pH 3.7–4.0), both manufactured with *S. thermophilus* as the predominant CSC LAB species, ranged from 550 to 800 mg/100 g and 1,200 to 1,700 mg/100 g, respectively [46],[58]. Consequently, a complete growth inhibition followed by a strong “tailing” survival of *L. monocytogenes* was found in Galotyri cheese [53] as well as in the present CSC-containing TGCM and RT-curds (Figure 1) which resembled an acid-curd cheese environment of pH 4.2–4.5 (Figure 2).

The type of CSC, the intensity of milk thermization and the initial level of artificial listerial contamination of TGCM appeared to also affect the survival and growth of *L. monocytogenes* as well as the % prevalence rates of the NisA+ *L. lactis* subsp. *cremoris* costarter. Indeed, contrary to the results in Figure 1, an inoculated (ca. 4 log CFU/mL) cocktail of the *L. monocytogenes* strains ISS G79, ISS G185 and Scott A increased more than 10-fold in coculture (37 °C for 6 h) with another CSC in ewes'/goats' milks previously thermized at 63 °C for 30 s [49]. Thus a 10-fold higher milk contamination with *L. monocytogenes* and 100-fold higher levels of background microbiota in the milder (63 °C) thermized milk, lowered the antagonistic effects of the CSC while the growth capacity of *L. monocytogenes* increased. That similar type of CSC also reduced the prevalence of the NisA+ strain M104 (counterpart of strain M78) at 3.1% compared to 81.2% when strain M104 was cultured in its absence in the same thermized milk samples at 37 °C for 6 h. The prevalence of strain M104 also fell from 81.2 to 11.9% when it was cocultured with the multiple Ent+ *E. faecium* KE82, both inoculated at a level of 6 log CFU/mL in thermized milk at 63 °C [49]. Thus higher levels of background microbiota, particularly antagonistic enterococci such as strain KE82 [51], also restricted the growth of the NisA+ strain M104. Conversely, the lowered levels of background microbiota in the present cheese milks thermized at 65 °C favoured the CSC *S. thermophilus* to predominate and minimize growth of *L. monocytogenes.* Growth of strain M78 in the absence of the CSC was also enhanced considerably in TGCM. Thus the faster and greater the growth of CSC *S. thermophilus*, the stronger the growth suppression of *L. monocytogenes* was anticipated, irrespective of background microbiota. Under the conditions of this study, however, the strongest suppression of *L. monocytogenes* by this particular CSC was not achieved unless a “balanced” portion of the indigenous raw milk microbiota survived to remain active in TGCM postthermally (Figure 1). An early growth (2–3 logs) of this natural microbiota, consisting of Ent+ enterococci, staphylococci and enterobacteria, was “prerequisite” for the minimization of *L. monocytogenes* growth by the primary inhibitor, the CSC, in factory-scale prepared TGCM and cooked curds. This microbiota grew at similar population levels in the corresponding Graviera cheeses within 24 h after manufacture, while it appeared to be partially responsible for major reductions in the residual nisin A activity in the CSC + M78 cheeses after fermentation [50]. Further research studies based on simplification approaches of the complex antilisterial microbial consortia in cooked hard cheese [59] are in progress in our laboratory to elucidate “hidden” interactions and biochemical activities of the background microbiota that assisted the CSC in suppressing *L. monocytogenes* growth completely. Probably this microbiota acted synergistically with the CSC by growing at the expense of milk nutrients other than those utilized by the CSC strains, and probably strain M78 [60],[61]. Conversely, in the CSC-SM there was no indigenous bacterial competition for the above nutrients, possibly in favour of the inoculated *L. monocytogenes* which utilized them. So the pathogen could increase 50-fold before the predominant CSC growth and its associated acid/low pH stress, or potentially L-lactic in undissociated form [21],[56], suppress its growth [49],[62]. The possibility that indigenous milk enzymes might have acted synergistically to increase the CSC inhibitory effects in TGCM can't be excluded, but this hypothesis requires further investigations also.

## 4. Conclusions

The interacting hurdle factors that minimized growth of *L. monocytogenes* during processing of cooked Graviera cheese milks were also the responsible set factors that concurrently reduced growth and counteracted the *in situ* NisA+ antilisterial effects of the novel costarter strains *L. lactis* subsp. *cremoris* M78, and its counterpart M104 strain, in the present and previous studies. The rapid dominant growth and accelerated milk acidifying capacity of the competitive CSC *S. thermophilus* in the 65 °C/TGCM during early process operations at its optimal growth temperature range (34 to 48 °C) were the primary hurdles against the pathogen and the costarter simultaneously. The surviving background microbiota in TGCM had an implicit assistant role, which has yet to be elucidated. In the absence of the CSC, the NisA+ strain M78 predominated in the 65 °C/TGCM, and thereby, its strongest *in situ* nisin A-mediated effects resulted in the highest inactivation rates of *L. monocytogenes* after 24 to 72 h at 22 °C. Thus, the wild NisA+ strains M78 and M104 are ideal LAB for use as novel primary starter cultures in traditional Greek soft, acid-curd cheeses, such as the PDO Galotyri cheese, which can be produced without thermophilic starters, particularly *S. thermophilus.* However, the present survival data of *L. monocytogenes* in the TGCMs and curds confirmed that this pathogen is exceptionally tolerant to cheese-related stresses and has the capacity of “tailing” survival patterns, without growth of its dormant cells, in acidified milks, acid-curd and fully ripened (cooked hard) cheese matrixes. Specifically, we showed the ability of *L. monocytogenes* to survive quite well at cultured milk pH values as low as 4.2 to 4.8 for up to 5 days. The pathogen survived even in the presence of nisin A and/or the prevalence of viable NisA+ M78 cells in its surrounding acidic milk environment, and thus it can't be easily eliminated during cheese processing.
